# Genetic and Phenotypic Basis of Autosomal Dominant Parkinson's Disease in a Large Multi-Center Cohort

**DOI:** 10.3389/fneur.2020.00682

**Published:** 2020-07-28

**Authors:** Suzanne Lesage, Marion Houot, Graziella Mangone, Christelle Tesson, Hélène Bertrand, Sylvie Forlani, Mathieu Anheim, Christine Brefel-Courbon, Emmanuel Broussolle, Stéphane Thobois, Philippe Damier, Franck Durif, Emmanuel Roze, François Tison, David Grabli, Fabienne Ory-Magne, Bertrand Degos, François Viallet, Florence Cormier-Dequaire, Anne-Marie Ouvrard-Hernandez, Marie Vidailhet, Ebba Lohmann, Andrew Singleton, Jean-Christophe Corvol, Alexis Brice, for the French Parkinson disease Genetics Study Group(PDG)

**Affiliations:** ^1^Sorbonne Université, Unité Mixte de Recherche (UMR) 1127, Paris, France; ^2^Unité de Recherche U1127 à l'Institut National de la Santé et de la Recherche Médicale (INSERM), Paris, France; ^3^Unité de Recherche Unité Mixte de Recherche (UMR) 7225 au Centre National de la Recherche Scientifique (CNRS), Paris, France; ^4^Institut du Cerveau (ICM), Paris, France; ^5^Institut de la Mémoire et de la Maladie d'Alzheimer (IM2A), Centre d'Excellence sur les Maladies Neurodégénératives (CoEN), Assistance Publique – Hôpitaux de Paris (AP-HP), Département de Neurologie, Groupe Hospitalier Pitié-Salpêtrière, Université Paris 6, Paris, France; ^6^Centre d'Investigation Clinique Pitié Neurosciences CIC-1422, Paris, France; ^7^Département de Neurologie aux Hôpitaux Universitaires de Strasbourg, Strasbourg, France; ^8^Institut de Génétique et de Biologie Moléculaire et Cellulaire (IGBMC), Illkirch, France; ^9^Fédération de Médecine Translationnelle de Strasbourg (FMTS), Université de Strasbourg, Strasbourg, France; ^10^Service de Pharmacologie Clinique, Faculté de Médecine, Hôpital Universitaire, Toulouse, France; ^11^Service de Neurologie B8, Hôpital Pierre Paul Riquet, Hôpital Universitaire, Toulouse, France; ^12^Université de Lyon, Institut des Sciences Cognitives Marc-Jeannerod, Unité Mixte de Recherche (UMR) 5229, Centre National de la Recherche Scientifique (CNRS), Bron, France; ^13^Hospices Civils de Lyon, Hôpital Neurologique Pierre-Wertheimer, Département de Neurologie C, Bron, France; ^14^Université de Lyon, Faculté de Médecine Lyon-Sud Charles-Mérieux, Oullins, France; ^15^Centre Hospitalier Universitaire de Nantes, Centre d'Investigation Clinique, Nantes, France; ^16^Département de Neurologie A, Centre Hospitalier Universitaire de Clermont-Ferrand, Clermont-Ferrand, France; ^17^Département de Neurologie, Groupe Hospitalier Pitié-Salpêtrière, Paris, France; ^18^Institut des Maladies Neurodégénératives, Centre Hospitalier Universitaire et Université de Bordeaux, Bordeaux, France; ^19^Centre de Neuroimagerie de Toulouse, Université de Toulouse - Institut National de la Santé et de la Recherche Médicale (INSERM) - Université de Toulouse, Toulouse, France; ^20^Centre des Neurosciences, Hôpital Universitaire de Toulouse, Toulouse, France; ^21^Unité de Neurologie, Hôpital Universitaire Avicenne, Hôpitaux Universitaires de Paris-Seine Saint Denis, Assistance Publique – Hôpitaux de Paris (AP-HP), Sorbonne Paris Nord, Bobigny, France; ^22^Equipe Dynamique et Physiopathologie des Réseaux Neuronaux, Centre pour la Recherche Interdisciplinaire en Biologie, Collège de France, Centre National de la Recherche Scientifique (CNRS) Unité Mixte de Recherche (UMR) 7241, Institut National de la Santé et de la Recherche Médicale (INSERM) U1050, Labex MemoLife, Paris, France; ^23^Département de Neurologie, Centre Hospitalier Intercommunal d'Aix-Pertuis, Aix-en-Provence, France; ^24^Laboratoire Parole et Langage, Unité Mixte de Recherche (UMR) 7309, Centre National de la Recherche Scientifique (CNRS) et Université d'Aix-Marseille, Aix-en-Provence, France; ^25^Centre Hospitalier Universitaire de Grenoble, Service de Neurologie, Grenoble, France; ^26^Department of Neurodegenerative Diseases, Hertie Institute for Clinical Brain Research, University of Tübingen, Tübingen, Germany; ^27^Laboratory of Neurogenetics, National Institute on Aging, National Institutes of Health, Bethesda, MD, United States

**Keywords:** Parkinson's disease, *LRRK2*, G2019S, *SNCA*, *VPS35*, autosomal dominant inheritance, genotype-phenotype correlations

## Abstract

*LRRK2, SNCA*, and *VPS35* are unequivocally associated with autosomal dominant Parkinson's disease (PD). We evaluated the prevalence of *LRRK2, SNCA*, and *VPS35* mutations and associated clinical features in a large French multi-center cohort of PD patients. Demographic and clinical data were collected for 1,805 index cases (592 with autosomal dominant inheritance and 1,213 isolated cases) since 1990. All probands were screened with TaqMan assays for *LRRK2* Gly2019Ser. In the absence of this mutation, the coding sequences of the three genes were analyzed by Sanger sequencing and/or next-generation sequencing. The data for the three genes were analyzed according to age at onset, family history, ethnic origin and clinical features. We identified 160 index cases (8.9%) with known pathogenic variants: 138 with pathogenic *LRRK2* variants (7.6%), including 136 with the Gly2019Ser mutation, 19 with *SNCA* point mutations or genomic rearrangements (1.1%), and three with the *VPS35* Asp620Asn mutation (0.16%). Mutation frequencies were higher in familial than isolated cases, consistent with autosomal dominant inheritance (12.0 vs. 7.3%; OR 1.7, 95% CI [1.2–2.4], *p* = 0.001). PD patients with *LRRK2* variants were more likely to have higher rates of late-onset PD (>50 years; OR 1.5, 95% CI [1.0–2.1], *p* = 0.03), whereas those with *SNCA* mutations tended to have earlier age at onset disease (≤ 50 years, *p* = 0.06). The clinical features of *LRRK2* carriers and those without any pathogenic variants in known PD-associated genes were similar. The likelihood of detecting disease-causing mutations was higher in cases compatible with autosomal dominant inheritance.

## Introduction

Heterozygous sequence variants of *LRRK2* or *VPS35*, and mutations or genomic rearrangements in *SNCA* cause monogenic Parkinson's disease (PD) with autosomal dominant (AD) inheritance [reviewed in (1)]. Only seven of the hundred or so variants of *LRRK2* reported to date (Asn1437His, Arg1441Gly/Cys/His, Tyr1699Cys, Gly2019Ser, and Ile2020Thr), which appear to be clustered in functionally important regions highly conserved throughout evolution, have been demonstrated to be pathogenic on the basis of co-segregation with the disease and absence or rarity in specific control populations (2). The most common of these mutations, *LRRK2* Gly2019Ser, has a reported frequency of 0% to above 40%, depending on the population considered (3), mostly due to a common founder effect (4). *SNCA* mutations are the second most common cause of autosomal dominant inherited PD; genomic duplications have been detected in ~1–2% of families with AD PD. Other *SNCA* mutations, such as whole-locus triplications and a few missense mutations (Ala53Thr/Glu/Val, Glu46Lys, Ala30Pro, and Gly51Asp), are extremely rare [reviewed in (1)]. Finally, *VPS35* was the first PD-causing gene to be identified by next-generation sequencing (NGS) in large multi-incident families (5, 6). Subsequent studies in multiple ethnic groups, including a large multi-center study, indicated that Asp620Asn was the only pathogenic variant, with a relative frequency ranging from 0.1 to 1% in familial PD, depending on population background (7). As the phenotype of AD PD closely resembles that of idiopathic PD, we assumed that rare variants of genes causing AD PD might also contribute to the etiology of isolated PD in the French population.

In this study, we aimed at determining the relative frequencies of known mutations of three genes, *LRRK2, SNCA*, and *VPS35*, in a large cohort of familial and isolated cases. The high prevalence of the *LRRK2* Gly2019Ser mutation provided us with a unique opportunity to compare in details clinical characteristics between carriers of this mutation and patients with no known mutations of PD-associated genes.

## Materials and Methods

### Patients

In total, 673 PD patients from 592 families and 1,213 isolated cases without known consanguinity were recruited from 1990 onwards, through the French Parkinson Disease Genetics Network (the PDG group, Supplementary Material). All participants underwent a detailed medical and family history, and a family tree were drawn. Familial cases compatible with AD inheritance, and referred to here as AD PD cases, were defined as AD cases with at least one other affected relative in a different generation, identified by an examination of secondary cases (*n* = 146) or on the basis of family history (*n* = 446). PD was diagnosed according to the clinical diagnostic criteria of the UK Parkinson Disease Society Brain Bank (PDSBB) (8). Comprehensive standardized interviews and neurological examinations were performed by movement disorder experts. Motor and non-motor symptoms were assessed by evaluating Unified Parkinson Disease Rating Scale (UPDRS) scores, Hoehn and Yahr staging, autonomic dysfunction, sleep, cognitive [Mini Mental State Examination (MMSE)], neuropsychological and behavioral scores. Early onset was defined as the onset of symptoms before the age of 51 years.

Informed consent was obtained from all participants, and genetic studies were approved by local ethics committees.

Most index cases were of European ancestry (*n* = 1,530; 84.8%), mostly French (*n* = 1,202; 78.6%); the others were North African (*n* = 221; 12.2%) or of other origins, including Asian, Sub-Saharan African or “mixed” origins (*n* = 42; 2.3%); or of unknown origin (*n* = 12; 0.7%). This study included 226 index cases from families with PD compatible with AD inheritance reported elsewhere (9, 10).

### Methods

Genomic DNA was obtained from peripheral blood lymphocyte or saliva samples (Oragene^TM^ DNA Self-Collection Kit, DNA Genotek), by standard protocols. Patients with variants of the *GBA* risk factor (*n* = 153), or with variants of genes for which the causal role in AD PD was uncertain, such as *GIGYF2* (*n* = 6), *EIF4G1* (*n* = 2), and *c9ORF72* (*n* = 4), were not included in this study. In addition, we excluded 25 (23 with bi-allelic *PRKN* mutations and two with bi-allelic *PINK1* mutations) of the 1,089 PD index cases who have been screened for autosomal recessive (AR)-PD associated genes [814 by gene panel/exome sequencing (see below) and 275 by direct sequencing of the two most frequent AR PD genes, *PRKN* and *PINK1*].

All index cases were genotyped in duplicate for *LRRK2* Gly2019ser, by the TaqMan allelic discrimination Assay-By-Design method, in accordance with the manufacturer's instructions, with 8 ng of DNA mixed with the TaqMan Genotyping Master Mix (Thermo Fisher Scientific Inc.) and custom-produced TaqMan SNP genotyping assays [C_63498123_10 (rs34637584), Thermo Fisher Scientific Inc.] on an Applied Biosystems PRISM 7000 sequence detection system (Thermo Fisher Scientific Inc.) or LightCycler® 480 machine (Roche, Life Technologies SAS). All patients found not to carry *LRRK2* Gly2019ser were then screened for pathogenic variants of the coding sequences of *LRRK2, SNCA*, and *VPS35*, by Sanger sequencing (*n* = 855), targeted sequencing of a customized next-generation sequencing (NGS) gene panel containing the 22 most prevalent PD-associated genes (*n* = 404; Supplementary Table 1), or available whole-exome sequencing (*n* = 410), as previously described (11, 12). We considered known pathogenic mutations of the three genes.

Sanger sequencing was used to confirm variants and co-segregation analyses were performed, where possible. *SNCA* rearrangements were detected by semi-quantitative multiplex PCR (13) or by the SALSA multiplex ligation-dependent probe amplification method (MLPA, MRC Holland, Amsterdam, the Netherlands; http://www.mlpa.com), according to the manufacturer's instructions.

### Statistical Analysis

Demographic and clinical characteristics are expressed as means and standard deviations for continuous variables and as counts and percentages for qualitative variables, separately for each group (i.e., with and without mutation). These characteristics were compared between patients with the *LRRK2* Gly2019ser mutation (*LRRK2*+) and those with no mutations of genes known to be associated with PD (genetically undefined PD), in Welch's *t*-tests for continuous variables and Fisher's exact tests for qualitative variables.

We used generalized linear models (GLMs) to compare clinical features between patients with the *LRRK2* Gly2019ser mutation and those with genetically undefined PD, with adjustment for sex, age at onset (AAO), and disease duration. Disease duration was not included in models of clinical features at onset. We used GLMs with identity links and normal distributions for continuous clinical features, and GLMs with logit links and Bernoulli distributions for binary clinical features. Fisher type II tests were performed to test each effect, and effect size was estimated with Cohen's f^2^. We corrected for multiple testing by the Benjamini-Hochberg method. Residual normality and heteroskedasticity were checked visually. Influencers and outliers were checked by calculating hat values and Cook distances. Only patients with no missing data for the covariables included in the models, such as AAO, sex, and disease duration, were retained for analysis. Statistical analyses were performed with R 3.6.1.

## Results

### Demographic and Clinical Data

Table 1 shows the baseline demographic and clinical characteristics of the 1,805 PD index cases included in the study. Men (*n* = 1,106, 61.3%) and early AAO cases (mean 47.4 [SD 12.5]; range 9–86 years; Table 1) were overrepresented, particularly among isolated cases (mean 46.1 [*SD* 12.5]; range: 9–79 years) as opposed to familial cases (mean 50.0 [*SD* 12.1]; range 10–86 years).

**Table 1:** Baseline demographics for our study population.

	**All index cases**	**AD PD index cases**	**Isolated cases**
	***n* = 1,805**	***n* = 592**	***n* = 1,213**
**Sex**, ***n*** **(%)**			
Male	1,106 (61.3)	342 (57.8)	764 (63)
Female	699 (38.7)	250 (42.2)	449 (37)
**Ancestry**, ***n*** **(%)**			
Europeans	1,530 (84.8)	525 (88.6)	1,005 (82.8)
North-Africans	221 (12.2)	55 (9.3)	166 (13.7)
Other/mixed origins	42 (2.3)	11 (1.9)	31 (2.6)
Unknown origins	12 (0.7)	1 (0.2)	11 (0.9)
Age at onset, (*SD*)	47.4 (12.5)	50.0 (12.1)	46.1 (12.5)
Range, years	9–86	10–86	9–79
Age at examination, (*SD*)	55.8 (13.3)	57.9 (12.5)	54.9 (13.5)
Range, years	12–88	16–88	12–85
Early-onset (≤ 50 years), *n* (%)	1,065 (62.7)	311 (54.6)	754 (66.8)
Late-onset (>50 years), *n* (%)	634 (37.3)	259 (45.4)	375 (33.2)
Disease duration, (*SD*)	8.4 (6.8)	7.8 (6.7)	8.8 (7.4)
Range, years	0–52	0–52	0–46

*Frequencies were compared in Fisher's exact tests for qualitative traits and means were compared in Welch's t-tests for continuous variables. Age-at-onset was missing for 106 index cases*.

*AD, autosomal dominant; PD, Parkinson's disease; SD, standard deviation*.

### Summary of the Mutations Identified

We identified seven different known mutations of *LRRK2, SNCA*, and *VPS35* in 160 of the 1,805 PD index cases (8.9%, 95% confidence interval (CI): [7.6–10.2]). With the exception of rare cases with the homozygous *LRRK2* Gly2019Ser mutation (*n* = 6), all the known mutations and gene multiplications identified were heterozygous. We found that 136 of the 138 *LRRK2* mutation carriers (7.5% of index cases, 95% CI [6.4–8.9]) carried the Gly2019Ser mutation; 13 of these patients had already been reported elsewhere (9). Two index cases carried the rare *LRRK2* Arg1441His mutation (9). *SNCA* mutations were found in 19 index cases (1.1%, 95% CI [0.63–1.6]), including 11 families already reported (13–15): one with whole-gene triplications, 14 with duplications, one with the Gly51Asp mutation, and three with the Ala53Thr mutation. The three PD index cases with the only *VPS35* mutation identified, Asp620Asn (0.17%) were described in a previous report (10).

The *LRRK2* Gly2019Ser mutation was more common in PD index cases of North-African origin (100/221, 45.2%; 95% CI [38.7–51.8]) than in Europeans (36/1,530, 2.4%; 95% CI [1.7–3.2]) (Fisher's exact test: odds ratio (OR) = 34.3, 95% CI [22.0–53.8], *p* < 0.0001; Table 2). By contrast, *SNCA* point mutations and locus triplications/duplications were found mostly in PD index cases of European ancestry, accounting for 1.2% (18/1,530) of such patients, whereas only one case of North-African ancestry (0.45%, 1/221) carried an *SNCA* duplication. *VPS35* mutations were identified exclusively in patients of European origin, accounting for 0.20% (3/1,530) of these individuals.

**Table 2:** Overall frequencies of *LRRK2, SNCA*, and *VPS35* mutations according to index case ethnicity, family history of Parkinson's disease and age at onset.

	**Europeans**	**North-Africans**	**North-Africans vs. Europeans OR, 95% CI, *p***	**AD PD**	**Isolated cases**	**AD PD vs. isolated cases OR, 95% CI, *p***	**EO-PD**	**LO-PD**	**LO-PD vs. EO-PD OR, 95% CI, *p***
	*n* = 1,530	*n* = 221		*n* = 592	*n* = 1,213		*n* = 1,063	*n* = 636	
All *LRRK2, n* (%, 95% CI)	38 (2.5%, [1.8–3.4])	100 (45.2%, [38.6–52.0])	*OR* = 32.5, [21.4–49.2], *p* < 0.0001	54 (9.1%, [6.9–11.7])	84 (6.9%, [5.6–8.5])	*OR* = 1.3, [0.9–1.9], *p* = 0.11	71 (6.7%, [5.3–8.4])	61 (9.6%, [7.4–12.2])	*OR* = 1.5, [1.0–2.1], *p* = 0.03
Gly2019Ser *n* (%)	36 (2.4%)	100 (45.2%)		52 (8.8%)	84 (6.9%)		71 (6.7%)	59 (9.3%)	
Arg1441His *n* (%)	2 (0.13%)	0 (0%)		2 (0.34%)	0 (0%)		0 (0%)	2 (0.32%)	
All *SNCA, n* (%, 95% CI)	18 (1.2%, [0.7–1.9])	1 (0.45%, [0.01–2.5])	*OR* = 0.38, [0.05–2.9], *p* = 0.50	14 (2.4%, [1.3–3.9])	5 (0.41%, [0.1–1.0])	*OR* = 5.9, [2.1–16.3], *p* = 0.0003	16 (1.5%, [0.9–2.4])	3 (0.47%, [0.1–1.4])	*OR* = 0.31, [0.09–1.1], *p* = 0.06
Triplications *n* (%)	1 (0.065%)	0 (0%)		1 (0.17%)	0 (0%)		1 (0.094%)	0 (0%)	
Duplications *n* (%)	13 (0.85%)	1 (0.5%)		11 (1.9%)	3 (0.25%)		11 (1.0%)	3 (0.47%)	
Ala53Thr *n* (%)	3 (0.20%)	0 (0%)		1 (0.17%)	2 (0.16%)		3 (0.28%)	0 (0%)	
Gly51Asp *n* (%)	1 (0.065%)	0 (0%)		1 (0.17%)	0 (0%)		1 (0.094%)	0 (0%)	
*VPS35* Asp620Asn, *n* (%, 95% CI)	3 (0.20%, [0.04–0.57])	0 (0%)	*p* = 1	3 (0.51%, [0.1–1.5])	0 (0%)	*p* = 0.04	2 (0.19%, [0.02–0.7])	1 (0.16%, [0.0–0.9])	*OR* = 0.84, [0.08–9.2], *p* = 1
Total mutations, *n* (%, 95% CI)	59 (3.9%, [2.9–4.9])	101 (45.7%, [39.0–52.5])	*OR* = 21.0, [14.5–30.4], *p* < 0.0001	71 (12.0%, [9.5–14.9]	89 (7.3%, [5.9–9.0])	*OR* = 1.7, [1.2–2.4, *p* = 0.001	89 (8.4%, [6.8–10.2]	65 (10.2%, [8.0–12.8]	*OR* = 1.3, [0.9–1.8], *p* = 0.22

*Age at onset was missing for 106 cases, including 6 with the LRRK2 G2019S mutation. We also considered patients with missing AAO data examined at an age ≤ 50 years (n = 26) to have early-onset PD*.

*AD, autosomal dominant; CI, confidence intervals; EO, early-onset; LO, late-onset; OR, odds ratio; PD, Parkinson's disease*.

Overall, mutations were more frequently identified in familial (71/592, 12.0%; 95% CI [9.5–14.9]) than in isolated cases (89/1,213, 7.3%; 95% CI [5.9–9.0], Fisher's exact test: OR 1.7, 95% CI [1.2–2.4], *p* = 0.001), particularly for *SNCA* (2.4%, 14/592 *vs*. 0.41%, 5/1,213, Fisher's exact test: OR 5.9, 95% CI [2.1–16.3], *p* = 0.0003; Table 2). An analysis of PD cases according to age at onset (≤ 50 years vs. >50 years) showed that *LRRK2* mutations were more frequent among late-onset PD cases (61/636, 9.6%; 95% CI [7.4–12.2] vs. 71/1063, 6.7%; 95% CI [5.3–8.4], Fisher's exact test: OR 1.5, 95% CI [1.0–2.1], p = 0.03; Table 2). By contrast, *SNCA* mutation carriers tended to have an earlier AAO (16/1063, 1.5% vs. 3/636, 0.5%, *p* = 0.06).

### Clinical Characteristics of Mutation Carriers and Comparison of *LRRK2* Gly2019Ser Mutation Carriers (*LRRK2+*) With Individuals With No Known PD Mutations (Genetically Undefined PD)

Co-segregation analyses identified 193 PD patients as mutation carriers: 151 with *LRRK2* Gly2019Ser and five with Arg1441His mutations, 29 with *SNCA* (see below) and eight with *VPS35* Asp620Asn mutations.

#### SNCA

The clinical characteristics of the 29 PD patients carrying either *SNCA* rearrangements [triplications (*n* = 2) and duplications (*n* = 21)] or missense mutations [Ala53Thr (*n* = 3) and Gly51Asp (*n* = 3)] are shown in Table 3. All but three of the families concerned originated from France. The remaining three families, originating from Italy, Turkey and Morocco, all had *SNCA* duplications. Within this cohort, *SNCA* duplications were the most frequent mutation identified (14/19, 73.7%), followed by the Ala53Thr mutation (3/19, 18.8%). Disease onset occurred earliest in patients with the Ala53Thr mutation (mean 34.7 [*SD* 7.6], range 26–40 years).

**Table 3:** Summary of the clinical data for patients carrying *SNCA, VPS35*, and *LRRK2* Arg1441His mutations identified in this study.

	***SNCA*** **rearrangements**		***SNCA*** **missense mutations**		***VPS35***	***LRRK2***
	**Triplications^**a**^**	**Duplications^**a**^**	**Ala53Thr**	**Gly51Asp^**b**^**	**Asp620Asn^**c**^**	**Arg1441His^**d**^**
No. of carriers (index cases)	2 (1)	21 (14)	3 (3)	3 (1)	8 (3)	5 (2)
No. of index cases with family history of PD	1/1	11/14 (78.6%)	1/3 (33.3%)	1/1	3/3 (100%)	2/2 (100%)
Sex (M:F)	2:0	7:14	1:2	1:2	5:3	2:3
Mean age at onset (*SD*) [range], years	42 (8.5) [36–48]	45.3 (6.3) [36–56]	34.7 (7.6) [26–40]	42.0 (15.7) [31–60]	57.1 (10.7) [38–71]	52.6 (9.6) [39–64]
Mean age at examination (*SD*) [range], years	50 (11.3) [42–58]	52.2 (6.0) [43–64]	42.7 (17.5) [28–62]	45.7 (18.7) [32–67]	65.0 (10.1) [52–80]	57.9 (10.0) [42–66]
Mean disease duration (*SD*) [range], year	8 (2.8) [6–10]	6.3 (3.9) [1–16]	8.2 (12.0) [0.5–22]	3.7 (3.1) [1–7]	7.9 (5.4) [1–17]	5.2 (5.1) [2–14]
**Signs at onset**						
Akinesia	1/2 (50%)	14/18 (77.8%)	3/3 (100%)	2/2 (100%)	7/7 (100%)	4/5 (80%)
Tremor	2/2 (100%)	6/18 (33.3%)	1/3 (33.3%)	0/2 (0%)	3/8 (37.5%)	1/4 (25%)
Micrographia	0/2 (0%)	8/18 (44.4%)	1/2 (50%)	1/2 (50%)	3/6 (50%)	2/5 (40%)
Dystonia	0/2 (0%)	0/17 (0%)	0/3 (0%)	0/2 (0%)	0/8 (0%)	1/4 (25%)
**Clinical signs at examination**						
Tremor	2/2 (100%)	11/19 (57.9%)	2/3 (66.7%)	1/3 (33.3%)	6/8 (75%)	4/4 (100%)
Bradykinesia	2/2 (100%)	19/19 (100%)	3/3 (100%)	3/3 (100%)	8/8 (100%)	5/5 (100%)
Rigidity	2/2 (100%)	19/19 (100%)	3/3 (100%)	3/3(100%)	8/8 (100%)	5/5 (100%)
Asymmetry	1/2 (50%)	16/17 (94.1%)	3/3 (100%)	3/3 (100%)	8/8 (100%)	5/5 (100%)
Apraxia	NA	0/16 (0%)	0/3 (0%)	0/2 (0%)	0/8 (0%)	0/5 (0%)
Dysarthria	NA	3/15 (20%)	1/3 (33.3%)	0/2 (0%)	0/8 (0%)	0/3 (0%)
Mean (or value) UPDRS III OFF (/108) (*SD*) [range], year	51 (7.1) [47, 56]	40.7 (23.7) [5–86]	47.7 (20.4) [30–70]	13	25 (9.9) [18–32]	23.8 (23.3) [2–56]
Mean (or value) UPDRS III ON (/108) (*SD*) [range], year	44.5 (2.1) [43–46]	17.7 (13.2) [4–48]	34 (1.4) [33–35]	7	21.9 (8.1) [8–33]	10.5 (9.5) [0–19]
Mean (or value) Hoehn and Yahr ON (/5) (*SD*) [range], year	NA	1.9 (0.64) [1–3]	3	1.5	2.3 (0.5) [2–3]	1.3 (0.3) [1–1.5]
**Treatment and its complications**						
Levodopa responsiveness^#^	0/2 (0%)	15/15 (100%)	2/3 (66.7%)	Mild to moderate	5/5 (100%)	5/5 (100%)
Dyskinesias	NA	9/15 (60%)	2/3 (66.7%)	2/3 (66.7%)	2/8 (25%)	1/4 (25%)
Motor fluctuations	NA	8/15 (53.3%)	2/3 (66.7%)	2/3 (66.7%)	3/8 (37.5%)	3/4 (75%)
Dystonia	NA	3/15 (20%)	0/3 (0%)	1/3 (33.3%)	0/8 (0%)	2/4 (50%)
**Non-motor signs**						
Cognitive impairment (MMSE <24/30)	1/2 (50%)	3/16 (18.8%)	0/3 (0%)	0/2 (0%)	0/8 (0%)	0/5 (0%)
Dysautonomia^*^	2/2 (100%)	6/16 (37.5%)	1/3 (33.3%)	0/2 (0%)	2/8 (25%)	2/5 (40%)
Depression/neuropsychiatric disorders	NA	4/16 (25%)	1/3 (33.3%)	2/3 (66.7%)	0/8 (0%)	0/5 (0%)

*Patients previously reported by*.

a *Ibanez et al. (13) and Books et al. (15)*.

b *Lesage et al. (14)*.

c *Lesage et al. (10)*.

d *Lesage et al. (9)*.

# *Levodopa responsiveness was defined as a >30% improvement in subjective perceived motor symptoms*.

* *Dysautonomia included at least one of the following three signs: orthostatic hypotension, erectile dysfunction, and/or urinary problems*.

*AD, autosomal dominant; MMSE, Mini Mental State Examination; NA, not available; PD, Parkinson's disease; UPDRS III, the motor subsection of the Unified Parkinson's Disease Rating Scale*.

Patients carrying the Ala53Thr mutation had an extra-pyramidal parkinsonian syndrome, but with heterogeneity between patients with the same mutation: Patient 1172-001, with both *SNCA* Ala53Thr and a heterozygous *PRKN* Thr240Met variant, had atypical PD, with a poor response to levodopa, early motor fluctuations and cerebellar signs. He rapidly developed impulse control disorders. This patient currently displays no cognitive decline. He had a bilateral subthalamic deep brain stimulation (STN-DBS). He received clozapine treatment for delusions with a beneficial effect. Patient 1219-001 presented a parkinsonian syndrome that responded well to levodopa, but developed severe dysarthria. She underwent unilateral internal globus pallidus (GPi)-DBS. This patient presented no major cognitive and behavioral signs other than an alteration of executive functions. A third PD patient, 196-016 presented early-onset (26 years) typical PD that responded well to levodopa. Detailed clinical data are provided in Supplementary Table 2.

Both *SNCA* locus triplications and Gly51Asp mutation were associated with early-onset atypical parkinsonism (mean AAO: 42.0 years [*SD* 8.5], range: 36–48 years and mean AAO: 42.0 [*SD* 15.7], range: 31–60 years, respectively). The patients with *SNCA* triplications were characterized by severe cognitive impairment in one of two carriers, dysautonomia, a poor response to levodopa in both patients. The three Gly51Asp mutation carriers had shorter disease duration (mean: 3.7 years [*SD* 3.1], range: 1–7 years), a mild-to-moderate response to levodopa, frequent psychiatric symptoms but no dementia or autonomic dysfunction (Supplementary Table 2). By contrast, AAO was highest in patients with *SNCA* duplications (mean AAO: 45.3 years [*SD* 6.3], range: 36–56 years), consistent with typical PD and a good response to levodopa (100%), with more than 50% of these patients reporting levodopa-induced motor complications. Non-motor symptoms, including depression/psychosis and dysautonomia, were present in about one third of the reported cases, but cognitive decline was less frequent (18.8%, 3/16). Detailed clinical characteristics for PD patients with *SNCA* multiplications are provided in Supplementary Table 3.

#### VPS35

Five of the eight PD patients carrying *VPS35* Asp620Asn mutations have been described before (10). The three newly genotyped patients were relatives of patient 838–006 (Supplementary Table 4). Briefly, patients carrying *VPS35* mutations had features similar to those with idiopathic PD, with a mean AAO of ~57 years (range: 38–71 years, Table 3): all patients presented the classical triad, with akinesia as the predominant symptom at onset (100%), but a much lower frequency of tremor as an initial symptom (37.5%), a good response to levodopa (100%), with <37% of those treated developing dyskinesias and motor fluctuations, and a low rate of dysautonomia (2/8, 25%), with no cognitive or neuropsychiatric symptoms or atypical signs.

#### LRRK2

We compared the clinical features of the *LRRK2* G2019S+ PD patient (*LRRK2*+) group with those of PD patients with no mutations of known PD-associated genes, excluding subjects with missing data for the covariables included in the models, such as sex, AAO, and disease duration: 135/151 *LRRK2*+ and 1,552/1,693 PD patients without mutations were included in the final analysis (Table 4). The proportion of men was greater in the genetically undefined PD patient group than in the *LRRK2*+ group (61.1 vs. 51.9%, *p* = 0.04). The mean AAO of the *LRRK2* Gly2019Ser carriers was 4 years higher than that of non-carriers (*p* < 0.001). The Gly2019Ser carriers were more likely to be of North-African ancestry (*p* < 0.001) and to report a family history of PD (*p* < 0.02). They had a higher UPDRS III score during the “OFF” state and a higher Hoehn and Yahr score during the “ON” state than non-carriers, but these results were no longer significant after adjustment for AAO and disease duration. The frequencies of signs at onset and at examination, the degree of response to treatment, motor complications and non-motor signs, including cognitive impairment and autonomic dysfunction were similar in both groups.

**Table 4:** Comparison of demographic and clinical characteristics of patients with Parkinson's disease by *LRRK2* mutation status (*LRRK2* carriers vs. patients without mutations in known Parkinson's disease-associated genes).

	***LRRK2*** **G2019S*******+***** **and patients without mutations; unadjusted comparisons**			***LRRK2*** **G2019S** *****+***** **and patients without mutations; adjusted comparisons**		
	***LRRK2*+ *n* = 135 (8%)**	**Genetically undefined PD *n* = 1,552 (92%)**	***p*-value**	**Coefficient or OR (CIs) (reference: non-mutation carriers) ∫**	***p*-value**	***p*-value adjusted^**¥**^**
**Demographic characteristics**						
Sex (% male)	70/135 (51.9%)	949/1,552 (61.1%)	0.04^*^			
Age at onset (*SD*), years	51.6 (12.8)	47.3 (12.8)	<0.001^*^			
Age at examination (*SD*), years	60.6 (13.3)	55.7 (13.4)	<0.001^*^			
Disease duration (*SD*), years	9.0 (8.0)	8.4 (7.0)	0.32			
**Ancestry**			<0.001^*^			
European	43/135 (31.9%)	1,390/1,543 (90.0%)				
North-African	92/135 (68.1%)	110/1,543 (7.1%)				
Other/Mixed origins	0/135 (0.00%)	43/1,543 (2.8%)				
**Family history of PD**			0.02^*^			
AD PD	61/135 (45.2%)	536/1,552 (34.5%)				
Isolated cases	74/135 (54.8%)	1,016/1,552 (65.5%)				
**Clinical characteristics**						
Levodopa responsiveness^#^	69/77 (89.6%)	788/958 (82.3%)	0.12	1.76 [0.81;3.80]	0.13	0.44
**Symptoms at onset**						
Dystonia	8/101 (7.9%)	125/1,306 (9.6%)	0.72	0.88 [0.41;1.87]	0.74	0.78
Akinesia	57/108 (52.8%)	805/1,332 (60.4%)	0.13	0.75 [0.50;1.11]	0.15	0.44
Tremor	72/107 (67.3%)	809/1,350 (59.9%)	0.15	1.32 [0.87;2.01]	0.19	0.44
Micrographia	22/102 (21.6%)	454/1,314 (34.6%)	0.009^*^	0.50 [0.31;0.81]	0.003^*^	0.06
**Symptoms at examination**						
Bradykinesia	109/111 (98.2%)	1,369/1,411 (97.0%)	0.77	1.73 [0.41;7.26]	0.42	0.63
Rigidity	106/111 (95.5%)	1,333/1,406 (94.8%)	1.00	1.12 [0.44;2.85]	0.80	0.80
Tremor	89/111 (80.2%)	1,040/1,397 (74.4%)	0.21	1.32 [0.81;2.15]	0.25	0.50
Asymmetry	105/108 (97.2%)	1,313/1,366 (96.1%)	0.79	1.64 [0.50;5.40]	0.38	0.62
**Motor features**						
UPDRS III ON (/108) (*SD*)	19.5 (13.4)	18.7 (13.2)	0.57	−0.73 [−3.62;2.16]	0.62	0.76
UPDRS III OFF (/108) (*SD*)	38.9 (18.2)	32.5 (17.6)	0.02^*^	4.91 [−0.33;10.14]	0.07	0.44
Hoehn and Yahr ON (/5) (*SD*)	2.2 (1.0)	2.0 (0.9)	0.03^*^	0.13 [−0.06;0.31]	0.17	0.44
Hoehn and Yahr OFF (/5) (*SD*)	2.7 (1.1)	2.4 (1.0)	0.21	0.18 [−0.18;0.54]	0.33	0.59
**Motor complications**						
Dyskinesias	52/98 (53.1%)	537/1,164 (46.1%)	0.21	1.42 [0.90;2.25]	0.13	0.44
Motor fluctuations	58/98 (59.18%)	640/1,164 (55.0%)	0.46	1.18 [0.75;1.86]	0.48	0.66
Dystonia	27/98 (27.6%)	303/1,164 (26.0%)	0.72	1.11 [0.68;1.80]	0.68	0.77
**Non-motor features**						
Dysautonomia^*^	9/94 (9.6%)	111/1,214 (9.1%)	0.85	0.84 [0.40;1.75]	0.64	0.76
MMSE score (/30) (*SD*)	27.4 (3.7)	28.2 (3.2)	0.07	−0.56 [−1.41;0.28]	0.19	0.44

*Data are given as the mean ± standard deviation for continuous variables and as counts (percentages) for qualitative variables*.

*Welch's t-test was used to compare the groups for continuous variables and Fisher's exact test was used for binary variables. Coefficients for continuous clinical features and odds ratios (ORs) for binary clinical features, confidence intervals (CIs) and P-values were calculated from GLMs with mutation status, sex, age, and disease duration, for all variables except for onset variables, for which only mutation status, sex, and age at onset were added. Linear models were used for continuous variables; GLMs with logit link and Bernoulli distributions were used for binary variables*.

# *Levodopa responsiveness was defined as a >30% improvement in subjective perceived motor symptoms*.

* *Dysautonomia included at least one of the following three signs: orthostatic hypotension, erectile dysfunction, and/or urinary problems*.

*AD, autosomal dominant; MMSE, CI, Confidence Intervals; Mini Mental State Examination; OR, Odds Ratio; PD, Parkinson's disease; UPDRS III, the motor subsection of the Unified Parkinson's Disease Rating Scale*.

¥ *P corrected for multiple testing by the Benjamini-Hochberg procedure*.

* *p < 0.05*.

Clinical comparisons between heterozygous (*n* = 144) and homozygous (*n* = 7) Gly2019Ser mutation carriers revealed no significant differences in sex (men: 52.1 vs. 42.9%, *OR* 1.4, CI [0.31–6,7], *p* = 0.71), AAO (mean 51.4 [*SD* 12.1] vs. mean 53.7 [*SD* 11.2] years, *p* = 0.62), disease duration (mean 8.7 [*SD* 6.7] vs. 9.1 [*SD* 4.5] years, *p* = 0.87) or clinical presentation, but the number of homozygous carriers was small.

Unlike *LRRK2* Gly2019Ser carriers, all patients with the Arg1441His mutation were French and all reported a family history of PD. They had a shorter disease duration (mean: 5.2 years [*SD* 5.1], range: 2–14 years vs. mean 9.0 years [*SD* 8.0], range: 0.5–63 years), were more likely to develop an akinetic-rigid motor phenotype (80 vs. 53%), had a slightly better response to levodopa (100 vs. 90%), and an absence of cognitive and neuropsychiatric symptoms, but a similar mean age at onset (52.6 years [*SD* 9.6], range: 39–64 years vs. 51.4 years [*SD* 12.1], range: 29–86 years).

## Discussion

This is one of the largest national multi-center studies to investigate the frequency of variants of the three major genes unequivocally linked to AD PD—*LRRK2, SNCA*, and *VPS35*—and their associated phenotypes in a large cohort of >1,800 French and North African index PD cases. We report an overall mutation frequency of 8.9% across both populations, the *LRRK2* G2019S mutation being the most frequently identified variant (7.5%), particularly in familial rather than isolated cases. However, the frequency of mutations differed considerably between populations. We confirm here that the *LRRK2* Gly2019Ser mutation is the principal genetic cause of PD in our cases of North-African ancestry, reaching an overall frequency of 45% (100/221) and 62% (34/55) in familial cases. By contrast, this mutation was present at a much lower rate of 2.4% (36/1,530) in our native French PD cases. Our findings are consistent with those of previous multi-center studies (16). *SNCA* duplications were the second most common type of mutations, identified in 14 index cases (0.78%). *SNCA* duplication carriers were mostly of European ancestry, particularly French (93%), tended to be predominantly females, probably due to random or recruitment bias, and had a higher frequency of a family history of PD. We also identified three unrelated PD patients carrying the *SNCA* Ala53Thr mutation. Although generally rare, this mutation appears to be particularly common in the Italian and Greek populations, due to a founder effect (17, 18). Only four individuals without Greek or Italian ancestry have been reported to carry this mutation, in haplotypes different from those reported in Greek and Italian families (19–22). Additional haplotype analysis would determine the ancestral origin of our three French mutation carriers. Other rare known mutations were also identified in our study: *VPS35* Asp620Asn and *LRRK2* Arg1441His in three and two AD PD families, respectively. At least 25 *LRRK2* Arg1441His carriers, including those described here, have been reported to date [(3); www.mdsgene.org]. Most were Caucasian, and all but one case reported a family history of the disease. This pathogenic variant was not found in more than 6,000 healthy controls tested (23) and is very rare in the Genome Aggregation Database (GnomAD) (1/31,298 alleles); it is therefore very likely to be pathogenic. Consistent with this conclusion, the Arg1441His variant affects the same amino-acid residue as two other recurrent PD-causing mutations (Arg1441Cys and Arg1441Gly). Finally, previous haplotype analyses did not support the hypothesis of a common founder for the Arg1441His variant, instead suggesting that there might be a mutational hotspot. Following initial reports of the existence of several *VPS35* variants (5, 6), pathogenicity has been confirmed only for the Asp620Asn variant. Consistent with our findings, this recurrent mutation has been identified predominantly in families of Caucasian descent affected by AD PD. A meta-analysis of 21,824 PD patients from 15 case-control studies performed worldwide from 2011 to 2016 identified an overall mutation frequency of 0.12% (0.29% in familial cases and 0.023% in isolated cases) [reviewed in (24)], and an absence of this mutation from healthy controls and the GnomAD public database.

The clinical features of our *LRRK2* mutation carriers, whether heterozygous or homozygous, were indistinguishable from those of patients with no mutations in known PD-associated genes. These features overlapped those of typical, idiopathic PD. In our study, patients with the G2019S mutation had a mean AAO of ~52 years, a high proportion of patients with late AAO (>50 years), a good response to levodopa, a predominance of tremor as a first symptom of PD, about a quarter had cognitive impairment, about 10% had dysautonomia, but no other atypical signs after a mean disease duration of ~10 years. Although the clinical features of the *LRRK2* Gly2019Ser carriers compared with patients with idiopathic PD in literature are conflicting [meta-analysis in (25)], even for the same ethnic PD population [i.e., of North-African origin; (26–31)], our data are consistent with those of 724 *LRRK2* mutation carriers listed in the MDSGene database. Like *LRRK2* mutation carriers, *VPS35* Asp620Asn carriers had a phenotype very similar overall to that of idiopathic PD: absence of atypical signs, excellent levodopa response, normal cognition, and absence of neuropsychiatric features. However, the mean AAO of our patients appeared to be later (57 years) than that reported in a recent meta-analysis (51 years) (32), due to the presence of multiple affected relatives with a late onset of disease within the same family (see Supplementary Table 4). Lastly, *SNCA* mutation carriers had motor features similar to those of idiopathic PD, but an overall earlier AAO, a shorter disease course, a higher frequency of motor complications, a higher frequency of non-motor signs and symptoms (cognitive decline in 17%, autonomic dysfunction in 39%, and psychotic symptoms, and depression in 32%). Atypical signs have also been observed in rare PD patients carrying the *SNCA* Gly51Asp mutation (14). In this study, we also identified a rare known variant of *SNCA*, His50Gln, in the homozygous state. This variant has been described as a causal variant associated with late-onset PD, dementia, and dystonia (33, 34), but a revaluation in larger datasets of PD patients and controls, including the GnomAD database (23/282,808 alleles), provided no evidence of pathogenicity for this variant (35). However, interestingly, the 42 year-old female patient carrying the His50Gln variant in our cohort had clinical features similar to those observed in carriers of other types of *SNCA* mutation carriers. She presented an early AAO (32 years), an excellent response to levodopa, motor complications, akinetic-rigid parkinsonism, and dystonia, an absence of cognitive decline and neuropsychiatric symptoms, but the presence of autonomic dysfunction and atypical neurological signs, such as postural instability, REM sleep behavior disorder (RBD) and impulse control disorders (see Supplementary Table 2). However, in this study, the *SNCA* His50Gln was found using our customized gene panel and in absence of whole exome/genome sequencing to detect other possible pathogenic mutations, its pathogenicity remains inconclusive.

The principal strength of this national multi-center study is the large group of well-phenotyped and genotyped patients and family members recruited at the 16 different PDG centers, and the use of a standardized protocol, ensuring comparable, and consistent clinical data reporting and diagnoses at each center. This enabled us to refine the estimated prevalence of mutations in genes causing AD PD in France. We show that our population, although mixed, has a relatively high frequency of *SNCA, LRRK2*, and *VPS35* mutations. However, the clinical data were cross-sectional, most patients were European or North African, and our populations were biased toward EO cases.

In most instances, the phenotypes of cases due to AD PD mutations are indistinguishable from those of cases without mutations, demonstrating the need for genetic analysis for their identification. Gene-specific disease-modifying therapies are currently being developed and tested. More generalized genetic testing is therefore required in PD patients, to identify those most likely to benefit from personalized care (36).

## Data Availability Statement

The raw data supporting the conclusions of this article will be made available by the authors, without undue reservation.

## Ethics Statement

The studies involving human participants were reviewed and approved by INSERM, CCPPRB (Comité Consultatif de Protection des Personnes dans la Recherche Biomédicale) du Groupe Hospitalier Pitié-Salpêtrière, Paris, France. Written informed consent to participate in this study was provided by the participants' legal guardian/next of kin.

## Author Contributions

SL conceived, designed and organized the study, wrote the first draft, reviewed, and critically revised the manuscript. J-CC and AB conceived the project, reviewed, and critically revised the manuscript. MH contributed to the statistical analysis and critically revised the manuscript. GM, CT, HB, SF, MA, CB-C, EB, ST, PD, FD, ER, FT, DG, FO-M, BD, FV, FC-D, A-MO-H, MV, EL, and AS contributed to the execution of the research project and critically revised the manuscript. All authors contributed to the article and approved the submitted version.

## Conflict of Interest

The authors declare that the research was conducted in the absence of any commercial or financial relationships that could be construed as a potential conflict of interest.

## Abbreviations

AAO: age at onset
AD: autosomal dominant
*LRRK2*: leucine-rich repeat kinase 2
MMSE: mini mental state examination
PD: Parkinson's disease
*VPS35*: vacuolar protein sorting 35.
