# Supplementary material for: Genetic and Phenotypic Basis of Autosomal Dominant Parkinson's Disease in a Large Multi-Center Cohort
Source: Front Neurol. 2020 Jul 28;11:682. doi: 10.3389/fneur.2020.00682 (PMC7399219; doi:10.3389/fneur.2020.00682)
Supplement: Supplementary file 1 [file Data_Sheet_1.PDF]

Genetic and phenotypic basis of autosomal dominant Parkinson disease in a large multi-center cohort

Suzanne Lesage, Marion Houot, Graziella Mangone, Christelle Tesson, Hélène Bertrand, Sylvie Forlani, Mathieu Anheim, Christine Brefel-Courbon, Emmanuel Broussolle, Stéphane Thobois, Philippe Damier, Franck Durif, Emmanuel Roze, François Tison, David Grabli, Fabienne Ory-Magne, Bertrand Degos, François Viallet, Florence Cormier-Dequaire, Anne-Marie Ouvrard-Hernandez, Marie Vidailhet, Ebba Lohmann, Andrew Singleton, Jean-Christophe Corvol, Alexis Brice for the French Parkinson disease Genetics Study Group (PDG)†

The French clinicians' network for Parkinson's disease genetics (the PDG group) members:
Yves Agid (site investigator, Department for the Central Nervous System, Paris), Mathieu Anheim (site investigator, Department of Neurology, Strasbourg), Michel Borg (site investigator, Department of Neurology, Nice), Alexis Brice (site investigator, Department of Genetics and Cytogenetics, Paris), Emmanuel Broussolle (site investigator, Pôle des Spécialités Neurologiques, Lyon), Jean-Christophe Corvol (site investigator, Center for Clinical Investigations, Paris), Philippe Damier (site investigator, Department of Neurology, Nantes), Luc Defebvre (site investigator, Service de Neurologie et Pathologie du Mouvement, Clinique Neurologique, Hôpital Roger Salengro, Lille), Alexandra Dürr (site investigator, Department of Genetics and Cytogenetics, Paris), Franck Durif (site investigator, Department of Neurology A, Clermont-Ferrand), Jean Luc Houetto (site investigator, service de neurologie, CHU de Poitiers, Poitiers), Paul Krack (site investigator, Pôle Psychiatrie et Neurologie, Grenoble), Stephan Klebe (site investigator, Centre for Clinical Investigations, Paris), Suzanne Lesage (site investigator, ICM INSERM U1127, Paris), Ebba Lohmann (site investigator, Department of Genetics and Cytogenetics, Paris), Maria Martinez (site investigator, INSERM Unit 563, Toulouse), Graziella Mangone (site investigator, Centre for Clinical Investigations, Paris), Pierre Pollak (site investigator, Pôle Psychiatrie et Neurologie, Grenoble), Olivier Rascol (site investigator, Clinical Investigation Centre, Toulouse), François Tison (site investigator, Pôle des Neurosciences, Cliniques de Neurologie, Bordeaux), Christine Tranchant (site investigator, Department of Neurology, Strasbourg), Marc Vérin (site investigator, Department of Neurology, Rennes), François Viallet (site investigator, Department of Neurology, Aix-en-Provence), and Marie Vidailhet (site investigator, Department of Neurology, Paris).

Supplementary Table 1. The 22 candidate genes involved in PD and related to parkinsonism included in targeted next-generation sequencing in this study

Gene	OMIM number	Chromosome location	*Genomic Coordinates (GRCh38/hg19)*
**Recessive PD-associated genes**			
PRKN	602544	6q26	chr6: 161,347,416-162,727,801
PINK1	608309	1p36.12	chr1: 20,633,454-20,651,510
DJ-1	602533	1p36.23	chr1: 7,961,653-7,985,281
ATP13A2	610513	1p36.13	chr1: 16,985,957-17,011,971
PLA2G6	603604	22q13.1	chr22: 38,111,494-38,192,099
FBXO7	605648	22q12.3	chr22: 32,474,681-32,498,830
SYNJ1	604297	21q22.11	chr21: 32,628,758-32,731,246
DNAJC6	608375	1p31.3	chr1: 65,264,693-65,415,868
VPS13C	54832	15q22-2	chr15: 61,852,388-62,060,464
**Dominant PD-associated genes**			
SNCA*	163890	4q21	chr4*: 89,724,098-89,838,323*
LRRK2*	609007	12q12	chr12: 40,224,889-40,369,284
VPS35*	601501	16q11.2	chr16: 46,657,978-46,689,231
DNAJC13	614334	3q22.1	chr3: 132,417,659-132,539,031
UCHL1	191342	4p13	chr4: 41,256,880-41,268,428
EIF4G1	600495	3q27.1	chr3: 184,314,494-184,335,357
**Related Parkinsonism-associated genes**			
SPG11	610844	15q21.1	chr15: 44,562,695-44,663,677
TH	191290	11p15.5	chr11: 2,163,928-2,174,080
GCH1	600225	14q22.2	chr14: 54,842,004-54,902,823
POLG	607608	11p15.4	chr15: 89,316,304-89,334,794
PANK2	606157	20p13	chr20: 3,888,822-3,933,086
DCTN1	601143	2p13.1	chr2*:* 74,361,153-74,392,086
Risk factor for PD			
GBA	606463	1q21	chr1: 155,234,447-155,244,861

*The three genes causing autosomal dominant Parkinson’s disease included in this study are indicated in bold type-face

Supplementary Table 2. Clinical characteristics of patients with Parkinson's disease carrying heterozygous *SNCA* point mutations

**Patient**	**1172-001**	**1219-001**	**196-016**	**138-018***	**138-029***	**138-032***	**1452-001****
Genes	*SNCA/PRKN*	*SNCA*	*SNCA*	*SNCA*	*SNCA*	*SNCA*	*SNCA*
Variants	Ala53Thr/ Thr240Met	Ala53Thr	Ala53Thr	Gly51Asp	Gly51Asp	Gly51Asp	His50Gln[&]
Origin	France	France	France	France	France	France	France
Family history of PD	Spo	Spo	AD	AD	AD	AD	Spo
Sex	M	F	F	F	F	M	F
Age at onset (years)	38	40	26	60	35	31	32
Age at examination (years)	38	62	28	67	38	32	42
Disease duration (years)	0.5	22	2	7	3	1	10
**Signs at onset**							
Akinesia	+	+	+	NA	+	+	+
Tremor	+	-	-	NA	-	-	-
Micrographia	NA	+	-	NA	+	-	+
Dystonia	-	-	-	NA	-	-	+
**Signs at examination**							
Tremor	+	-	+	+	-	-	-
Bradykinesia	+	+	+	+	+	+	+
Rigidity	+	+	+	+	+	+	+
Asymmetry	+	+	+	+	+	+	+
Apraxia	-	-	-	NA	-	-	NA
Dysarthria	-	+	-	NA	-	-	+
UPDRS III OFF (/108)	43	70	30	NA	NA	13	NA
UPDRS III ON (/108)	35	33	NA	NA	7	NA	34
Hoehn & Yahr ON (/5)	NA	3	NA	NA	1.5	NA	3
**Treatment and its complications**							
LEDD (mg)	300	2500	250	NA	375	NA	516
Levodopa improvement (%)[#]	19	50	100	Mild	Mild	Moderate	60

Levodopa-induced dyskinesia	-	+	+	+	+	-	+
Motor fluctuations	+	+	-	+	-	+	+
Dystonia	-	-	-	-	+	-	+
**Non-motor signs**							
MMSE (/30)	28	> 24	> 24	NA	28	30	28
Dysautonomia	No	OH, ED, UP	No	NA	No	No	UP
Neuropsychiatric signs	Yes	No	No	Yes	Yes	No	No
**MRI**	Normal	Normal	Normal	Mild generalized atrophy	Mild generalized atrophy	NA	Normal

*Patients previously reported by Lesage *et al.*, 2013
**Patient previously reported by Blauwendraat et *al.*, 2018
[&]Variant now considered benign
[#]Levodopa responsiveness was defined as a >30% improvement in subjective perceived motor symptoms
AD, autosomal dominant; ED, erectile dysfunction; LEDD, levodopa equivalent daily dose; MMSE, Mini Mental State Examination. MRI, magnetic resonance imaging; NA, not available; OH, orthostatic hypotension; PD, Parkinson’s disease; Spo, sporadic; UP, urinary problems; UPDRS III, the motor subsection of the Unified Parkinson's Disease Rating Scale

Supplementary Table 3. Clinical characteristics of patients with Parkinson's disease carrying heterozygous *SNCA* duplications/triplications

**Patient**	**131-022***	**131-024***	**131-026***	**321-021***	**410-001***	**437-010***	**437-012***	**437-005***
	Dup	Dup	Dup	Dup	Dup	Dup	Dup	Dup
Origin	France	France	France	France	Italy	France	France	France
Family history of PD	AD	AD	AD	AD	AD	AD	AD	AD
Sex	M	F	F	F	F	F	F	F
Age at onset (years)	48	40	50	46	50	38	38	54
Age at examination (years)	57	56	51	54	56	46	43	56
Disease duration (years)	9	16	1	8	6	8	5	2
**Signs at onset**								
Akinesia	+	-	+	+	+	+	+	+
Tremor	+	+	-	-	-	+	-	-
Micrographia	-	-	-	-	-	+	-	-
Dystonia	NA	-	-	-	-	-	-	-
**Signs at examination**								
Tremor	+	+	-	-	-	+	+	+
Bradykinesia	+	+	+	+	+	+	+	+
Rigidity	+	+	+	+	+	+	+	+
Asymmetry	+	+	+	-	+	+	+	NA
Apraxia	-	-	NA	-	-	-	NA	NA
Dysarthria	-	+	NA	-	+	-	NA	NA
UPDRS III OFF (/108)	55	86	5	32	35	33	47	NA
UPDRS III ON (/108)	NA	NA	NA	5	NA	13	17	18
Hoehn & Yahr ON (/5)	NA	NA	NA	2	2.5	2.5	NA	1.5
**Treatment and its complications**								
LEDD (mg)	NA	12	NA	1100	300	50	500	NA

Levodopa improvement (%)#	NA	>30	NA	40	>30	60	67	40
Levodopa-induced dyskinesia	NA	+	NA	+	-	+	+	-
Motor fluctuations	NA	+	NA	+	-	+	-	-
Dystonia	NA	-	NA	-	+	+	+	-
**Non-motor signs**								
MMSE (/30)	26	26	NA	27	> 24	> 24	NA	22
Dysautonomia	No	No	NA	ED, UP, OH	OH	No	NA	ED, UP
Neuropsychiatric signs	No	No	NA	Yes	No	No	NA	No
**MRI**	Post-traumatic hypodense internal frontal lesion	NA	NA	Normal	Normal	Normal	NA	NA

Continued

**Patient**	**652-169***	**947-005***	**1164-001***	**1117-001***	**652-171**	**947-003**	**539-006**	**539-007**	**1423-001**
	Dup	Dup	Dup	Dup	Dup	Dup	Dup	Dup	Dup
Origin	Turkey	France	France	France	Turkey	France	France	France	France
Family history of PD	AD	AD	AD	AD	AD	AD	AD	AD	Spo
Sex	M	F	F	M	F	F	F	M	M
Age at onset (years)	NA	43	56	39	51	36	56	NA	39
Age at examination (years)	57	45	62	50	56	45	64	58	45
Disease duration (years)	NA	2	6	11	5	9	8	NA	6
**Signs at onset**									
Akinesia	NA	-	+	-	NA	+	+	NA	+
Tremor	NA	+	-	-	NA	-	-	NA	+
Micrographia	NA	+	+	-	NA	+	+	NA	-
Dystonia	NA	-	-	-	NA	+	-	NA	-
**Signs at examination**									
Tremor	NA	-	+	+	-	-	-	NA	+
Bradykinesia	NA	+	+	+	+	+	+	NA	+
Rigidity	NA	+	+	+	+	+	+	NA	+
Asymmetry	NA	+	+	+	NA	+	+	NA	+
Apraxia	NA	-	-	-	NA	-	-	NA	-
Dysarthria	NA	-	+	NA	NA	-	-	NA	-
UPDRS III OFF (/108)	NA	NA	57	NA	NA	NA	NA	NA	48
UPDRS III ON (/108)	NA	32	48	NA	NA	12	4	NA	32
Hoehn & Yahr ON (/5)	NA	3	2	NA	NA	2	1	NA	2.5
**Treatment and its complications**									
LEDD (mg)	NA	560	1200	NA	600	277	687.5	NA	1200
Levodopa	NA	70	> 30	> 30	NA	70	> 30	NA	50

improvement (%)[#]									
Levodopa-induced dyskinesia	NA	-	+	NA	+	+	+	NA	+
Motor fluctuations	NA	-	+	NA	+	+	+	NA	+
Dystonia	NA	-	-	NA	-	-	-	NA	-
**Non-motor signs**									
MMSE (/30)	NA	> 24	23	< 24	NA	> 24	30	NA	> 24
Dysautonomia	NA	ED, UP	No	ED, UP, OH	NA	No	No	NA	ED, UP
Neuropsychiatric signs	NA	No	Yes	No	NA	Yes	No	NA	Yes
**MRI**	NA	Normal	Stroke sequelae	Cortical and sub-cortical atrophy	NA	Normal	Normal	NA	Normal

Continued

**Patient**	**1737-001**	**1746-001**	**1333-005**	**1255-001**	**14-010***	**14-011***
	Dup	Dup	Dup	Dup	Trip	Trip
Origin	France	France	France	Morocco	France	France
Family history of PD	Spo	Spo	AD	AD	AD	AD
Sex	M	F	M	F	M	M
Age at onset (years)	45	40	45	46	36	48
Age at examination (years)	50	51	46	48	42	58
Disease duration (years)	5	11	1	2	6	10
**Signs at onset**						
Akinesia	+	-	+	+	-	+
Tremor	-	+	-	-	+	+
Micrographia	+	-	+	+	-	-
Dystonia	-	-	-	-	-	-

**Signs at examination**						
Tremor	+	+	-	+	+	+
Bradykinesia	+	+	+	+	+	+
Rigidity	+	+	+	+	+	+
Asymmetry	+	+	+	+	+	-
Apraxia	-	-	-	-	NA	NA
Dysarthria	-	-	NA	NA	NA	NA
UPDRS III OFF (/108)	NA	NA	9	NA	47	56
UPDRS III ON (/108)	7	11	NA	13	43	46
Hoehn & Yahr ON (/5)	2	1	1	2	NA	NA
**Treatment and its complications**						
LEDD (mg)	NA	400	NA	NA	1200	800
Levodopa improvement (%)#	50	> 30	NA	45	NA	20
Levodopa-induced dyskinesia	-	-	NA	-	NA	NA
Motor fluctuations	-	-	NA	-	NA	NA
Dystonia	-	-	NA	-	NA	NA
**Non-motor signs**						
MMSE (/30)	29	24	> 24	> 24	24	6
Dysautonomia	No	No	No	No	ED, UP	ED, UP
Neuropsychiatric signs	No	No	No	No	NA	NA
**MRI**	NA	Normal	Normal	Normal	NA	NA

*Patients previously reported by Ibanez *et al.*, 2009 and Book et *al.*, 2018

#Levodopa responsiveness was defined as a >30% improvement in subjective perceived motor symptoms

AD, autosomal dominant; Dup, duplications; ED, erectile dysfunction; LEDD, levodopa equivalent daily dose; MMSE, Mini Mental State Examination. MRI, magnetic resonance imaging; NA: not available; OH, orthostatic hypotension; PD, Parkinson's disease; Spo, sporadic; Trip, triplications; UP, urinary problems; UPDRS III, the motor subsection of the Unified Parkinson's Disease Rating Scale

Supplementary Table 4. Clinical characteristics of patients with Parkinson's disease carrying the heterozygous *VPS35* Asp620Asn mutation

**Patient**	**142-001***	**142-003***	**142-006***	**258-001***	**858-006***	**858-003**	**858-004**	**858-005**
Origin	France	France	France	France	France	France	France	France
Family of PD	AD	AD	AD	AD	AD	AD	AD	AD
Sex	M	M	F	F	M	F	M	M
Age at onset (years)	48	59	63	38	53	67	71	58
Age at examination (years)	54	60	80	52	62	70	77	65
Disease duration (years)	6	1	17	14	9	3	6	7
**Signs at onset**								
Akinesia	+	+	-	+	+	+	+	+
Tremor	+	-	+	-	+	-	-	-
Micrographia	NA	+	-	-	-	-	+	+
Dystonia	-	-	-	-	-	-	-	-
**Signs at examination**								
Tremor	+	-	+	-	+	+	+	+
Bradykinesia	+	+	-	+	+	+	+	+
Rigidity	+	+	-	+	+	+	+	+
Asymmetry	+	+	NA	+	+	+	+	+
Apraxia	-	-	-	-	-	-	-	-
Dysarthria	-	-	-	-	-	-	-	-
UPDRS III OFF (/108)	32	18	NA	NA	NA	NA	NA	NA
UPDRS III ON (/108)	21	8	NA	17	28	33	25	21
Hoehn & Yahr ON (/5)	2	2	NA	3	3	2	2	2
**Treatment and its complications**								
LEDD (mg)	300	375	375	650	NA	125	300	700
Levodopa improvement (%)[#]	>30	55	>30	>30	NA	NA	50	NA

Levodopa-induced dyskinesia	-	-	-	+	-	-	-	+
Motor fluctuations	-	-	-	+	+	-	-	+
Dystonia	-	-	-	-	-	-	-	-
**Non-motor signs**								
MMSE (/30)	30	30	> 24	26	29	> 24	30	30
Dysautonomia	No	No	No	OH	ED, UP	No	No	No
Neuropsychiatric signs	No	No	No	No	No	No	No	No
**MRI**	NA	NA	NA	Normal	Normal	NA	NA	NA

*Patients previously reported by Lesage *et al.*, 2012

#Levodopa responsiveness was defined as a >30% improvement in subjective perceived motor symptoms

AD, autosomal dominant; ED, erectile dysfunction; LEDD, levodopa equivalent daily dose; MMSE, Mini Mental State Examination. MRI, magnetic resonance imaging; NA, not available; OH, orthostatic hypotension; PD, Parkinson’s disease; Spo, sporadic; UP, urinary problems; UPDRS III, the motor subsection of the Unified Parkinson's Disease Rating Scale

Supplementary Table 5. Clinical characteristics of patients with Parkinson's disease carrying the heterozygous *LRRK2* Arg1441His mutation

**Patient**	**221-010***	**221-013***	**221-015**	**221-009***	**412-012***
Origin	France	France	France	France	France
Family history of PD	AD	AD	AD	AD	AD
Sex	F	M	F	M	F
Age at onset (years)	52	49	39	59	64
Age at examination (years)	66	54	42	61	66
Disease duration (years)	14	5	3	2	2
**Signs at onset**					
Akinesia	+	+	+	-	+
Tremor	-	-	NA	-	+
Micrographia	-	+	+	-	-
Dystonia	-	+	NA	-	-
**Signs at examination**					
Tremor	+	+	+	+	+
Bradykinesia	+	+	+	+	+
Rigidity	+	+	+	+	+
Asymmetry	+	+	+	+	+
Apraxia	-	-	-	-	-
Dysarthria	-	NA	-	NA	-
UPDRS III OFF (/108)	24	56	2	NA	13
UPDRS III ON (/108)	5	19	NA	18	0
Hoehn & Yahr ON (/5)	1.5	1.5	NA	NA	1
**Treatment and its complications**					
LEDD (mg)	150	1500	400	300	NA
Levodopa	70	65	60	30	100

improvement (%)#					
Levodopa-induced dyskinesia	+	-	-	-	NA
Motor fluctuations	+	+	+	-	NA
Dystonia	+	+	-	-	NA
**Non-motor signs**					
MMSE (/30)	29	> 24	> 24	> 24	30
Dysautonomia	No	UP	ED, UP	No	No
Neuropsychiatric signs	No	No	No	No	No
**MRI**	Normal	Cavernous angioma	Abnormal	Hypersignals of the white matter	Normal

*Patients previously reported by Lesage *et al.*, 2009

#Levodopa responsiveness was defined as a >30% improvement in subjective perceived motor symptoms

AD, autosomal dominant; ED, erectile dysfunction; LEDD, levodopa equivalent daily dose; MMSE, Mini Mental State Examination. MRI, magnetic resonance imaging; NA: not available; PD, Parkinson’s disease; Spo, sporadic; UP, urinary problems; UPDRS III, the motor subsection of the Unified Parkinson's Disease Rating Scale
